# Equity trends for the UHC service coverage sub-index for reproductive, maternal, newborn and child health in Pakistan: evidence from demographic health surveys

**DOI:** 10.1186/s12939-023-02043-w

**Published:** 2023-11-02

**Authors:** Nabila Zaka, Maida Umar, Ahsan Maqbool Ahmad, Ikhlaq Ahmad, Tahira Ezra Reza, Mariyam Sarfraz, Faran Emmanuel

**Affiliations:** 1 UNICEF, Islamabad, Pakistan; 2 Health Services Academy, Islamabad, Pakistan; 3 Centre for Global Public Health, Islamabad, Pakistan; 4 University of Manitoba, Winnipeg, Canada

**Keywords:** Equity, Socio-economic status, RMNCH, UHC service coverage

## Abstract

**Background:**

Pakistan, the world's sixth most populous country and the second largest in South Asia, is facing challenges related to reproductive, maternal, newborn and child health (RMNCH) that are exacerbated by various inequities. RMNCH coverage indicators such as antenatal care (ANC) and deliveries at health facilities have been improving over time, and the maternal mortality ratio (MMR) is gradually declining but not at the desired rates. Analysing and documenting inequities with reference to key characteristics are useful to unmask the disparities and to amicably implement targeted equity-oriented interventions.

**Methods:**

Pakistan Demographic Health Survey (PDHS) based UHC service coverage tracer indicators were derived for the RMNCH domain at the national and subnational levels for the two rounds of the PDHS in 2012 and 2017. These derivations were subgrouped into wealth quintiles, place of residence, education and mothers’ age. Dumbbell charts were created to show the trends and quintile-specific coverage. The UHC service coverage sub-index for RMNCH was constructed to measure the absolute and relative parity indices, such as high to low absolute difference and high to low ratios, to quantify health inequities. The population attributable risk was computed to determine the overall population health improvement that is possible if all regions have the same level of health services as the reference point (national level) across the equity domains.

**Results:**

The results indicate an overall improvement in coverage across all indicators over time, but with a higher concentration of data points towards higher coverage among the wealthiest groups, although the poorest quintile continues to have low coverage in all regions. The UHC service coverage sub-index on RMNCH shows that Pakistan has improved from 45 to 63 overall, while Punjab improved from 50 to 59 and Sindh from 43 to 55. The highest improvement is evident in Khyber Pakhtunkhwa (KP) province, which has increased from 31 in 2012 to 51 in 2017. All regions made slow progress in narrowing the gap between the poorest and wealthiest groups, with particularly noteworthy improvements in KP and Sindh, as indicated by the parity ratio. The RMNCH service coverage sub-index gap was the greatest among women aged 15–19 years, those who belonged to the poorest wealth quintile, had no education, and resided in rural areas.

**Conclusions:**

Analysing existing data sources from an equity lens supports evidence-based policies, programs and practices with a focus on disadvantaged subgroups.

## Introduction

Pakistan, the world's sixth most populous country and the second largest in South Asia, is facing challenges related to reproductive, maternal, newborn and child health (RMNCH) that are exacerbated by various inequities [1, 2]. Pakistan's maternal mortality ratio of 186, neonatal mortality ratio of 42, and under five mortality rate of 74 are higher than those of neighbouring countries, indicating that Pakistan lags behind in terms of reproductive and child health outcomes [3, 4].

Health equity implies that every person has the opportunity to attain his or her full health potential, and no one is disadvantaged from achieving this potential because of social position or other socially determined circumstances [5, 6]. Several studies have shown that the causality of health inequity may lie in biological factors (age, sex), socioeconomic determinants of health or other variables such as ethnicity, language, education or minority status [6, 7]. Improving the equity and coverage of maternal, neonatal and child survival services are critical to reduce the overall maternal, neonatal and child health-related disease burden [8]. The World Health Organization (WHO) considers equity as an intermediate objective of universal health coverage (UHC) and defines UHC reforms as “reforms that ensure that health systems contribute to health equity, social justice and the end of exclusion, primarily by moving towards universal access and social health protection”. UHC and global health equity are considered the driving forces in achieving the Sustainable Development Goals (SDGs) [9]. Pursuing health equity in light of SDG targets focuses on eliminating health disparities strongly associated with social disadvantages and implies that all people can use health services when needed with sufficient quality and without facing any financial hardships [10, 11].

Pakistan, the world's sixth most populous country and the second largest in South Asia, is facing challenges related to reproductive, maternal, newborn and child health (RMNCH) that are exacerbated by various inequities [1, 2]. Pakistan's maternal mortality ratio of 186, neonatal mortality ratio of 42, and under five mortality rate of 74 are higher than those of neighbouring countries, indicating that Pakistan lags behind in terms of reproductive and child health outcomes [3, 4]. The current National Health Vision of Pakistan (2016–2025) places greater emphasis on health equity than has been historically done, with a focus on reducing urban‒rural disparities [12]. Access to essential health services and health equity are key challenges in a country of 230 million people, with an estimated 46 million people being economically disadvantaged and living below the poverty line. However, Pakistan has witnessed a reduction in consumption-based poverty from 50.4% to 24.3% (2005 vs 2015) and a decline in the multidimensional poverty index from 0.29 to 0.19 (2004 vs 2015) [13, 14].

Despite improvements in coverage of antenatal care (ANC), skilled birth attendance and immunization, healthcare disparities are a grim reality in Pakistan, and the gains have not benefitted all population subgroups judiciously [15, 16]. To achieve the broader goals of the UHC and RMNCH targets, health services must reach the most underserved segments of the population. As a first step towards achieving SDG targets, Pakistan needs to fill in the gaps in the current knowledge of baseline inequities in RMNCH at a subnational level. With this context, this study was conducted to assess the inequities in coverage of RMNCH interventions between 2012 and 2017 in Pakistan at the subnational level. The greater granularity of equity analysis and mapping equity trends over time will help public health planners and providers to identify the most disadvantaged subgroups of women and children who are being left behind. It will also provide a baseline against which future progress in the RMNCH subdomain can be ascertained to facilitate monitoring the impact of efforts to reduce inequities to achieve SDG 2030 targets.

## Methods

### Data sources

The Pakistan Demographic and Health Survey (PDHS) is a population-based, nationally representative cross-sectional survey that is conducted every five years. This survey uses a standardized questionnaire and employs a two-stage cluster sampling design. The first stage involves the selection of clusters with probability proportional to their size, and the second stage involves the systematic selection of a specific number of households. PDHS is considered to be one of the most comprehensive and reliable sources of data on health and demographic indicators at the national and subnational levels. The last PDHS was conducted in Pakistan in 2017–18, and we conducted a secondary analysis of data from the last two successive PDHSs conducted in 2012–13 [17] and 2017–18 [4]. These are the most recent data sets available in Pakistan at a national level for the indicators of interest. A total of 14,540 households were interviewed in 2017, including Azad Jammu and Kashmir and Gilgit Baltistan, compared to 12,943 in 2012–13, excluding Azad Jammu and Kashmir. The data are open access, and sample weights were used to compute various indicators.

### Data analysis

The UHC service coverage for the RMNCH subdomain includes four tracer indicators including demand for family planning satisfied with modern contraceptive methods, DPT3 immunization, antenatal care (at least four visits), and care-seeking for suspected pneumonia in the last two weeks preceding the survey [18]. These tracer indicators were measured at the national and subnational levels across the strata of wealth quintiles (ranked as poorest, poorer, middle, wealthier and wealthiest), place of residence (urban, rural), education level (illiterate, primary, middle, secondary, higher) and mothers’ age (< 20, 20–34, 35 and above). Dumbbell charts were created for RMNCH cluster tracer indicators to show the wealth quintile-specific coverages and inequities across the two time points.

The geometric mean of four primary component indicators was used to construct a composite *“UHC Service Coverage RMNCH sub-index”* [19, 20] at the national and using the following formula.

$${\text{UHC service coverage sub}-\text{index for RMNCH}\hspace{0.17em}=\hspace{0.17em}\left(\mathrm{FP}*\mathrm{ANC}4*\mathrm{DPT}*\mathrm{Pneumonia}\right)}^{1/4}$$

The parity indices were computed for the UHC Service Coverage sub-index to examine the trends and equity gaps at the national and subnational levels. The larger the gaps between the two groups of interest, the greater the parity index deviates from 1. Absolute and relative measures such as high to low absolute difference, population attributable risk and high to low ratio were applied to the UHC Service Coverage RMNCH sub-index to measure the gap between high and low performing provinces over time [21].

**Table:** 

Summary measure	Formula
Absolute difference	${y}_{h}-{y}_{l}$
Relative ratio	$\frac{{y}_{h}}{{y}_{l}}$
Population attributable risk	${y}_{h}-y$

where ${y}_{h}$ represents the high performing region; ${y}_{l}$ represents the low-performing region and y represents the national average. High to low absolute differences and high to low relative ratios demonstrate the gap between the high- and low-performing regions, while the population attributable risk estimates the overall population health improvement with the premise that all regions have the same level of health services as the reference point (national level) across the equity domains. The statistical analysis was carried out using R 4.1.3 and SPSS 22.0.

## Results

**Fig. 1:**
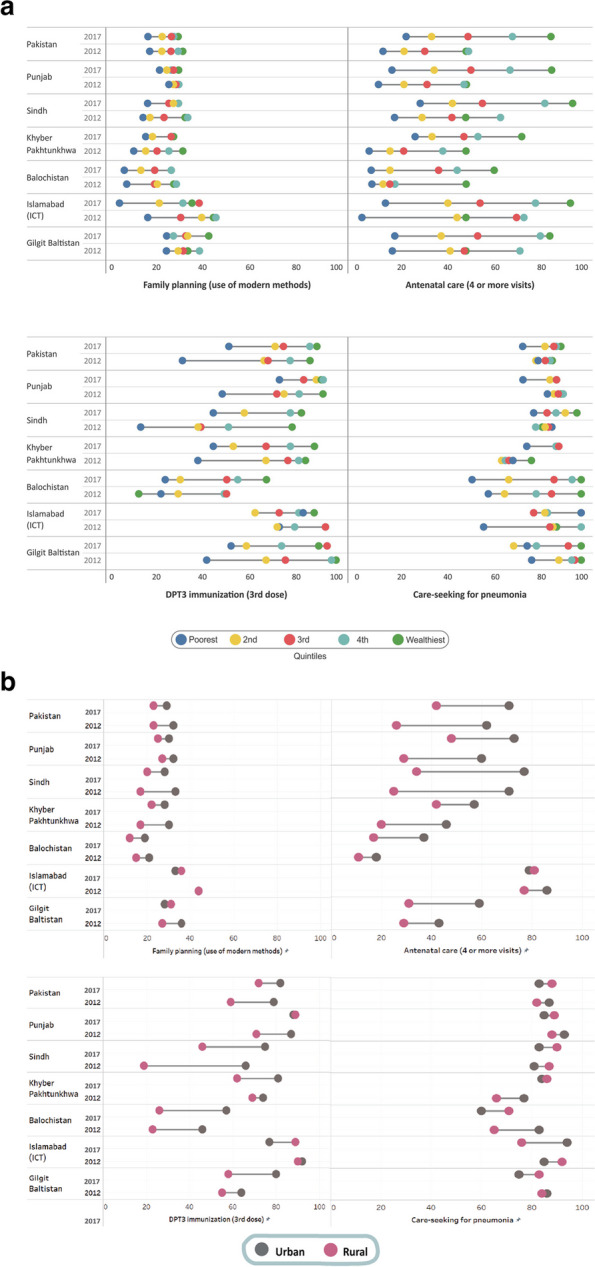
Equity trends of RMNCH health service coverage by (**a**) wealth quintiles and (**b**) place of residence at the national and subnational levels, 2012–17. Notes: Pakistan excludes Gilgit Baltistan and Azad Jammu & Kashmir. Care-seeking for pneumonia is the percentage of children (under 5 years) for whom advice or treatment was sought with symptoms of acute respiratory infection in the 2 weeks preceding the survey

Figure 1 shows the comparison of wealth quintile-specific and place of residence RMNCH service coverage estimates at the national and subnational levels during the years 2012 and 2017. Overall, it indicates an improvement in coverage across all indicators over time, but a higher concentration of data points towards higher coverage among the wealthiest groups, although the poorest quintile continues to have low coverage in all regions. A region wise comparison analysis shows that Balochistan has the lowest coverage in most of the indicators, followed by Gilgit, KP, Sindh and Punjab. The increase in coverage between 2012 and 2017 was noticed for antenatal care (4 or more visits) and DPT3 vaccination, while family planning and care seeking for suspected pneumonia had stagnant coverage within the quintiles and between the regions (Fig. 1a).

Urban Pakistan during 2017 has shown the utilization of family planning satisfied with modern contraceptive methods at 29%, while in rural areas, it was slightly lower at 23%. The antenatal care (4 or more visits) has high coverage in urban areas (71%), compared to rural areas (42%). The DPT3 immunization coverage achieved 82%, as compared to rural areas (72%). Seeking care for suspected pneumonia demonstrated a higher estimate of 88%, while rural areas reported a slightly lower rate of 82.9%. The analysis presented a consistent pattern where urban areas exhibit higher coverage estimates compared to rural areas across all four RMNCH tracer indicators with notable improvements over time. The urban–rural inequities are more apparent for antenatal care (4 or more visits) and DPT3 immunization. The rural areas, except ICT, have coverage lower than 50% and the lowest estimates in rural Balochistan (17%), followed by GB (31%) and Sindh (34%) in 2017. However, for family planning and care seeking for pneumonia, Punjab, KP and ICT have relatively fewer disparities depicting considerable progress over time (Fig. 1b).

**Fig. 2:**
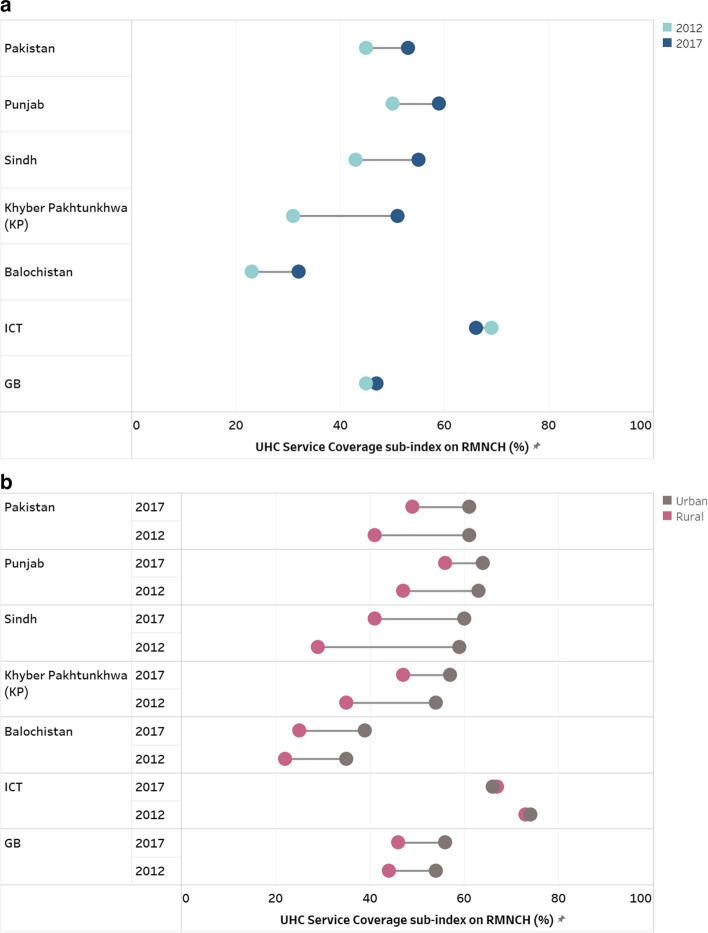
UHC Service Coverage sub-index on RMNCH at (**a**) national and subnational levels by (**b**) place of residence, 2012–17

UHC service coverage sub-index on RMNCH shows that Pakistan has improved from 45 to 63 overall, while Punjab improved from 50 to 59 and Sindh from 43 to 55. The highest improvement is evident in Khyber Pakhtunkhwa (KP) province, which has increased from 31 in 2012 to 51 in 2017. Islamabad Capital Territory (ICT) has shown a slight decrease in the sub-index which dropped from 69 to 66 due to a decrease in DPT3 vaccination coverage (Fig. 2a). The analysis illustrates a trend of disparities in UHC service coverage sub-index between urban and rural areas across different regions in Pakistan. The greatest disparities between urban and rural areas are pronounced in Sindh and Balochistan. (Fig. 2b).

Table 1 illustrates the changes in the parity ratio for the UHC service coverage subindex at the national and subnational levels between 2012 and 2017 across the considered equity strata. Pakistan has shown steady progress in reducing the gaps in the index among the poorest and wealthiest quintiles, place of residence and education levels. However, these gaps have increased among women less than 20 years of age as the parity ratio has declined from 0.69 to 0.65.

**Table 1:** Equity gaps in UHC Service Coverage sub-index on RMNCH at national and subnational levels, 2012–17

Region	Poorest to wealthiest disparity ratio		Rural to urban disparity ratio		Illiterate to highly educated disparity ratio		Maternal age disparity ratio between < 20 years and > 20 years	
	**2012**	**2017**	**2012**	**2017**	**2012**	**2017**	**2012**	**2017**
Pakistan^a^	0.42	0.52	0.68	0.81	0.57	0.62	0.69	0.65
Punjab	0.49	0.55	0.75	0.88	0.65	0.69	0.61	0.61
Sindh	0.35	0.53	0.49	0.68	0.49	0.59	0.75	0.76
KP	0.34	0.54	0.67	0.83	0.55	0.65	0.89	0.78
Balochistan	0.54	0.27	0.64	0.63	0.44	0.40	0.60	0.39
ICT	0.31	0.39	0.99	1.00	0.80	0.75	na	0.71
GB	0.48	0.48	0.81	0.82	0.55	0.50	0.80	na

^a^Pakistan excludes Gilgit Baltistan and Azad Jammu & Kashmir. Maternal age disparity ratio is not applicable for ICT and GB among women aged < 20 years seeking care for pneumonia due to a lack of cases reported in this age group during the study period

All regions made slow progress in narrowing the gap between the poorest and wealthiest groups, with particularly noteworthy improvements in KP and Sindh, as indicated by the parity ratio. The decrease in the parity ratio from 0.54 in 2012 to 0.27 in 2017 reflects an increase in the gap between the poorest and wealthiest quintiles in Balochistan. Similarly, the gaps have widened in Balochistan among women who are less than 20 years of age and illiterate. Among the provinces/regions, GB remained stagnant in terms of index across the quintiles and maternal age groups.

The magnitude of inequities using the subnational UHC service coverage sub-index on RMNCH is presented by the absolute difference and relative ratio between high and low performing regions (Table 2). Higher values reflect larger inequities between various groups. According to the high to low absolute difference, inequity between the low and high performing regions increased by almost 4% in 2017 and remained low in terms of coverage among all regions. The trend shows that rural areas had a higher degree of disparity urban regions. The RMNCH service coverage sub-index gap was greatest among women aged 15–19 years who belonged to the poorest wealth quintile, had no education, and resided in rural areas.

**Table 2:** Regional inequalities UHC Service Coverage sub-index on RMNCH, 2012–17

Background characteristics		Regional inequalities 2012		Regional inequalities 2017	
		**High to low absolute difference (percentage points)**	**High to low relative ratio**	**High to low absolute difference (percentage points)**	**High to low relative ratio**
**Wealth quintile**	Poorest	18.06	2.11	21.75	2.38
	Poorer	32.12	2.22	24.99	1.96
	Middle	30.04	1.86	20.79	1.49
	Wealthier	34.74	1.92	22.57	1.53
	Wealthiest	46.84	2.56	18.07	1.31
**Place of residence**	Urban	38.86	2.12	26.97	1.69
	Rural	50.78	3.28	41.92	2.69
**Mother's education**	Illiterate	40.14	2.88	28.14	2.13
	Primary	42.09	2.25	26.05	1.7
	Middle	25.24	1.57	14.44	1.29
	Secondary	31.86	1.79	26.22	1.53
	Higher	27.73	1.57	8.45	1.14
**Mother's age**	< 20	37.7	na	47.45	na
	20–34	46.36	2.84	37.32	2.31
	35–49	50.44	3.02	33.9	2.02

Maternal age disparity ratio is not applicable for ICT and GB among women aged < 20 years seeking care for pneumonia due to a lack of cases reported in this age group during the study period

**Fig. 3:**
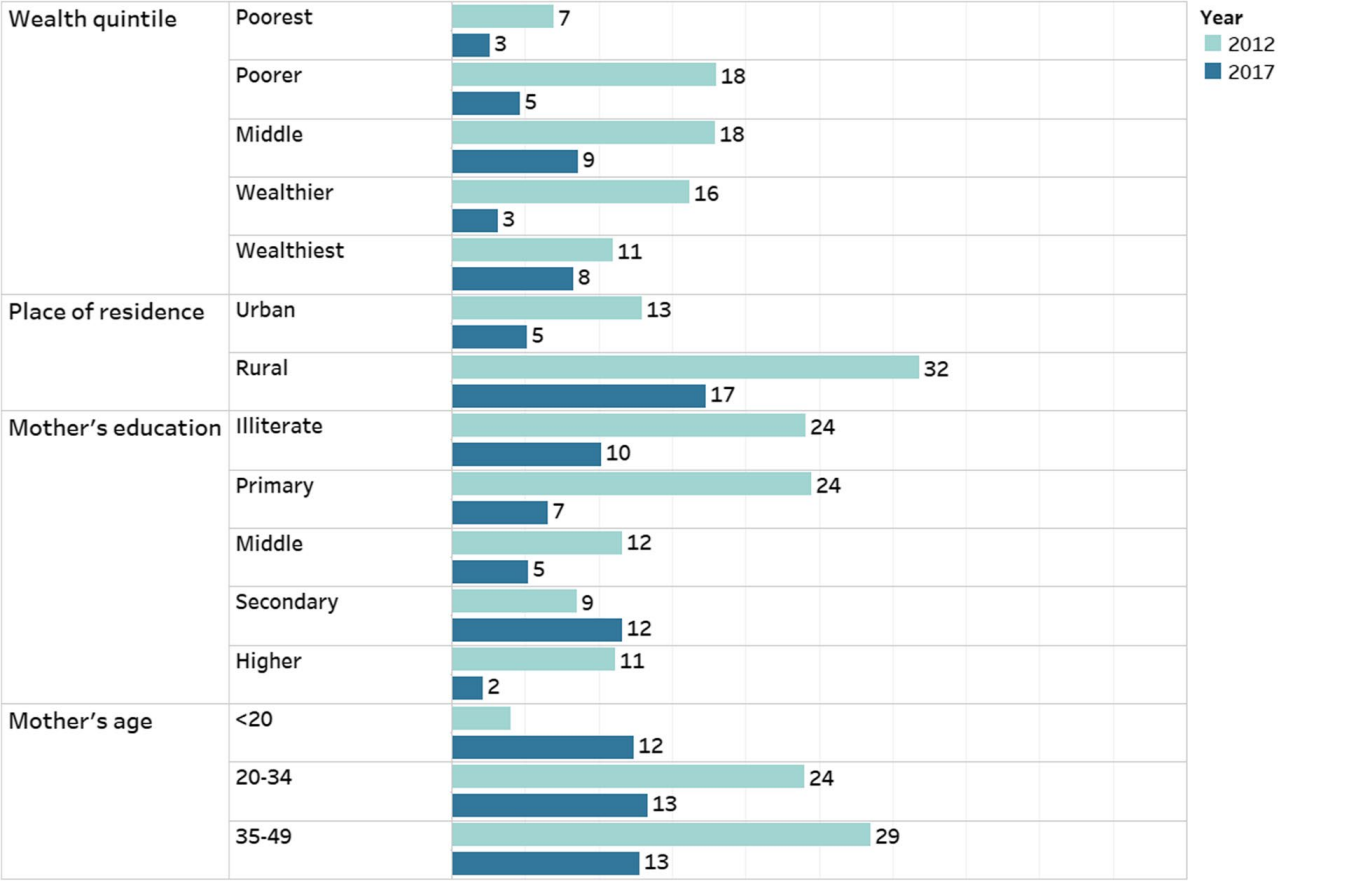
Population attributable risk, 2012–17

The attributable risk shows a higher degree of inequity among women aged < 20 years, illiterate women and women with secondary education, whereas it has decreased among women with higher education. A high differential was recorded among women with primary education, belonging to the age group of 35–49 years and rural residents across the two surveys. The population-attributable risk of the UHC service coverage sub-index for RMNCH over time (Fig. 3) effectively demonstrates how much regions can improve by eradicating inequities.

## Discussion

This study provides a comprehensive assessment of RMNCH service coverage at the national and subnational levels, revealing an overall improving trend in reproductive and maternal health coverage from 2012–2017. However, the pace of improvement varies among subgroups and provinces. While progress has been made in closing the coverage gap, the trajectory of improvement appeared to have slowed down for demand satisfied by modern methods and care-seeking for pneumonia [22–24]. Despite the improvements, persistent socioeconomic and urban‒rural inequalities persist across the RMNCH service indicators, posing challenges to achieving universal health coverage in Pakistan. These findings are consistent with previous research, emphasizing that coverage improvements are more pronounced among poor or rural populations, reflecting inequitable access to RMNCH services [24, 25].

The utilization of key maternal and child health services such as ANC, skilled birth attendance, and immunization have improved over time, but Pakistan has not been able to achieve the desired reduction in maternal, newborn, and child mortality rates [26]. This indicates the limited translation of the increase in service utilization into noticeable improvements in health outcomes. The concept of effective coverage, which is gaining recognition in the field of maternal and child health, highlights the importance of not only measuring service utilization but also assessing the tangible impact of these services on health outcomes [27, 28].

The disparities in Human Development Index (HDI) values between Balochistan and Punjab further illustrate the uneven development across provinces. With a significantly lower HDI value of 0.473 in Balochistan than in Punjab (0.572), it is evident that Balochistan lags behind in various development aspects, including healthcare services [29]. The findings from Fig. 1, which highlight the lowest coverage in Balochistan across most RMNCH indicators, support the notion of inadequate development and resource allocation in the region. The link between the disparities in poverty reduction and development indicators across provinces lies in the fact that socioeconomic factors and development indicators are closely intertwined with the availability and accessibility of healthcare services [30]. As elicited through our analysis, the RMNCH sub-index disparity ratio reflected higher inequities among women aged 15–19 years of age in the poorest quintile residing in rural areas. The women’s hierarchical level of education led to an incremental decrease in the risk of inequity.

There are notable variances and gaps in the health systems across the provinces of Pakistan, which contribute to disparities in access to and delivery of healthcare services. These gaps manifest in various ways, including differences in infrastructure, healthcare workforce distribution, availability of essential medical equipment and supplies, and healthcare financing. In comparison to the public sector, the private sector is relatively more established in Punjab, Sindh, and KP compared to Balochistan. The devolution of power from the federal to provincial governments has also posed challenges in terms of technical expertise and policy development, affecting the implementation and effectiveness of healthcare initiatives [31]. One of the key factors contributing to urban‒rural disparities is the unequal distribution of health services outlets and allied resources. Urban areas tend to have comparatively better infrastructure, a higher concentration of healthcare facilities, and a greater availability of healthcare professionals. Cultural and social factors also impact urban‒rural disparities in healthcare. The security situation in remote areas of some provinces has hindered the availability of skilled healthcare professionals, further exacerbating disparities and impeding progress. Differences in health-seeking behaviors, educational levels, and awareness about available healthcare services contribute to variations in healthcare utilization [32, 33]. These factors may result in delayed or inadequate care for all segments of the population.

Insufficient resources, inadequate infrastructure, and socioeconomic inequalities hinder the delivery and utilization of RMNCH services, leading to disparities in coverage across different regions and wealth quintiles. The surge in private facility-based deliveries [34] potentially associated with higher quality care causes financial burden for women and children due to increased out-of-pocket expenditures. However, current social protection programs such as the Benazir Income Support Programme (BISP), Sehat Sahulat, and Ehsaas present a unique opportunity to identify and target the most economically disadvantaged individuals and provide them with essential healthcare services [35]. Moreover, the assessment of the best and worst progressors based on the population attributable risk of the UHC service coverage sub-index in terms of equity dimensions provides valuable insights into the potential for improvement in different provinces of Pakistan. By examining the population attributable risk, we can identify that Punjab has made progress in reducing inequities and those that still need further improvement. Provinces with better performance in terms of equity dimensions serve as examples to learn from, as they have successfully addressed the underlying factors contributing to disparities in healthcare access and outcomes.

### Limitations

Due to the unavailability of recent data, establishing up-to-date provincial baselines becomes challenging. However, utilizing older data helps create a foundation reference for future progress. Furthermore, sampling techniques used in DHS surveys do not specifically capture information on wealth quintiles, mother's education, or age. The sample size can be impacted by the concentration of certain groups in geographical areas due to random cluster selection. While PDHS data are not representative at the district level, the Mixed Indicator Cluster Survey (MICS) data can be used for district-level analysis. However, comparability is a challenge in MICS, as information is not simultaneously available for all subnational regions. The available data do not allow real-time monitoring of changes in equitable coverage, which necessitates monitoring through the District Health Information System (DHIS) based on household characteristics.

## Conclusions

In conclusion, our study underscores the need to incorporate equity analysis as a routine component in monitoring UHC implementation and SDG reporting. By assessing interprovincial variations and utilizing the UHC index to prioritize districts for equity-focused programs, we can strengthen national monitoring efforts. To achieve health equity, sustained efforts are necessary to overcome barriers that hinder access to healthcare services and ensure equitable distribution of high-quality services. By addressing social determinants of health and tailoring interventions to meet the needs of disadvantaged populations, we can make substantial progress towards reducing health disparities.

Future research should focus on exploring potential solutions to address the aforementioned challenges, employing qualitative research methods to identify specific barriers to access and utilization. Moreover, integrating a quality of care dimension into the UHC RMNCH index and tracer indicators is crucial, as it will drive national initiatives to improve quality of care by stablishing global agreed-upon indicators for effective coverage.

## Data Availability

The data used in this study is publicly available at DHS website (www.dhsprogram.com). Other materials such as syntax files can be shared upon request to the corresponding author.
